# Multifocal Dysembryoplastic Neuroepithelial Tumour with Intradural Spinal Cord Lipomas: Report of a Case

**DOI:** 10.1155/2011/734171

**Published:** 2011-09-07

**Authors:** Richard D. White, Avinash K. Kanodia, Esther M. Sammler, John N. Brunton, Craig A. Heath

**Affiliations:** ^1^Department of Clinical Radiology, Ninewells Hospital and Medical School, NHS Tayside, Dundee DDI 9SY, UK; ^2^Department of Neurology, Ninewells Hospital and Medical School, NHS Tayside, Dundee DDI 9SY, UK

## Abstract

We report a case of temporal lobe epilepsy and incomplete Brown-Sequard syndrome of the thoracic cord. Computed tomography and magnetic resonance (MR) imaging showed multiple supratentorial masses with the classical radiological appearances of multifocal dysembryoplastic neuroepithelial tumour (DNET). Spinal MR imaging revealed intradural lipomas, not previously reported in association with multifocal DNET. Presentation and imaging findings are discussed along with classification and natural history of the tumour.

## 1. Introduction

Dysembryoplastic neuroepithelial tumour (DNET) is a predominantly intracortical, supratentorial tumour. It is categorized by the World Health Organisation as a grade 1 tumour (2007) in the neuronal and mixed neuronal-glial tumour group, along with such lesions as ganglioglioma, paraganglioma, and central neurocytoma [1]. DNET typically affects younger people and is usually confined to the temporal lobes and, as a recognized cause of intractable epilepsy, accounts for up to 8% of temporal lobe tumours resected for epilepsy [2].

Multifocality of DNET was first reported by Leung et al. in 1994 [3] and represents a much rarer clinical entity. To our knowledge, this is the eighth case of multifocal DNET, and the first to have concomitant spinal cord lipomas.

## 2. Case Report

A 20-year-old man presented with a few years history of complex partial seizures with increasing frequency and one episode of secondary generalization. He usually experienced symptoms of déjà-vu and visceral aura followed by short loss of awareness without automatisms. Recovery was gradual and accompanied by a right-sided headache. Seizure seminology was thought to be of temporal lobe origin. He reported reduced visual acuity on the right side since birth, mild left arm weakness, and problems when running due to tripping and stiffness. Nevertheless, he was physically very active. He was the product of an unremarkable pregnancy and spontaneous delivery. Developmental milestones were delayed from the age of 3 months onwards and he was extensively investigated because of irritability and floppiness at around the same time. No conclusive diagnosis was reached and all tests—in particular screening for infections—were unremarkable. He attended mainstream school until the age of 16, with poor performance. Family history was unremarkable and he had two healthy siblings. 

Examination revealed a right-sided iris coloboma, mild dysmorphism with an underdeveloped left upper limb and shoulder girdle, and also lumbar subcutaneous lipomas, but no other cutaneous features. Cranial nerve examination was unremarkable except for impaired vision in the right eye. He had mild left-sided pyramidal weakness with an MRC (medical research council) power grade of 4+/5, generalized hyperreflexia with accentuation on the left, and bilateral positive Babinski signs. There was reduced light touch and vibration sense up to hip level on the left and impaired pain and temperature sensation up to knee level on the right. There were no cerebellar, bladder, bowel, or erectile problems. Interictal electroencephalography demonstrated slow wave changes over both temporal regions. 

**Figure 1:**
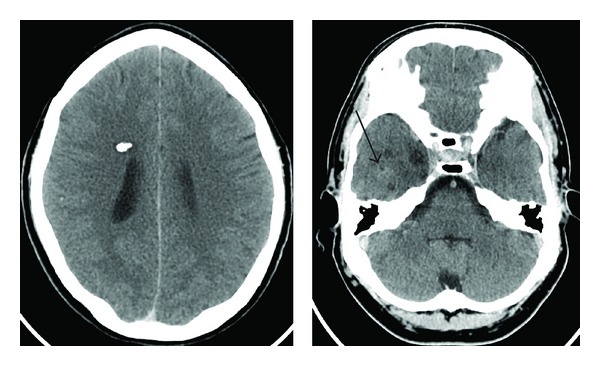
CT scan shows a dense calcified nodule in the subependymal aspect on right side and a poorly enhancing area in right temporal lobe (arrow).

**Figure 2:**
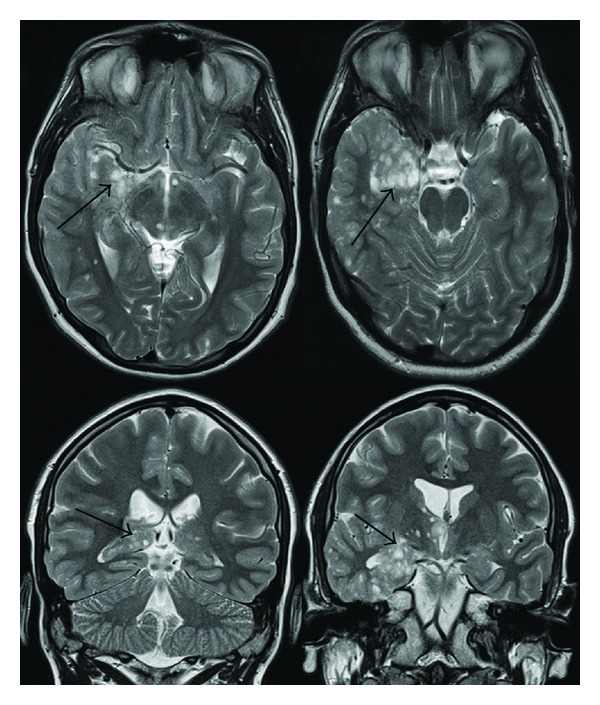
Axial and coronal T2 images demonstrating the right temporal lobe lesion (arrows) with bilateral numerous nodular lesions.

**Figure 3:**
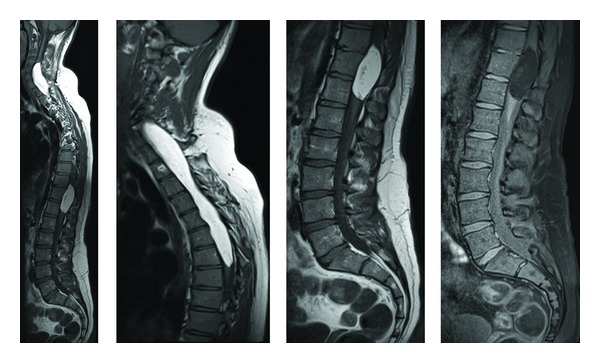
Sagittal T1 imaging of the spine with fat suppression (far right) demonstrating intradural spinal cord lipomas.

Computed tomography scanning revealed several abnormalities. A poorly enhancing heterogeneous lesion was evident in the right temporal lobe, and a densely calcified nodule was present in the subependymal region of the right frontal lobe (Figure 1). Subsequent magnetic resonance (MR) imaging (Figure 2) performed (T2-weighted images TR/TE-4300/114 ms, T1-weighted images TR/TE-500/7.8 ms) showed a large lobulated lesion within the right temporal lobe, mainly on its medial aspect, including hippocampus and amygdala. It exhibited a nodular and septated cystic appearance, with some peripheral enhancement and an area of low signal on T2 and T2* imaging, thought to represent calcification. Overlying cortical dysplasia was also evident. Similar lesions were present within the right thalamus and along the anterior commisure, with multiple further nodules within the right temporal lobe, deep frontal white matter and in upper right midbrain. Similar appearances were present on the left side, albeit to a much lesser extent. Large intramedullary spinal lesions were evident, one extending from the level of the C4 vertebra to T7 and another from T10 to T12, with fat signal on T1- and T2-weighted images and fat suppression (Figure 3), in keeping with spinal cord lipomas.

Imaging findings were classical for DNET, and considering other abnormalities, were highly suggestive of multifocal DNET. Multidisciplinary discussions between radiology, neurology, and neurosurgery teams and the patient resulted in the decision that biopsy or other surgery would not be undertaken due to the nonaggressive nature of the lesion and the minimal impact specified symptoms were having on the patient's quality of life.

## 3. Discussion

DNET was first proposed as a specific entity by Daumas-Duport et al. in 1988 [4], who coined the term after clinicopathological analysis of 39 young patients with tumour-associated intractable partial complex seizures (or, less commonly, headache). The authors identified features suggestive of a dysembryoplastic origin and highlighted that surgery was curative without the need for chemo- or radiotherapy. 

The precise pathogenesis of DNET remains unclear. One paper has expressed doubts as to the neoplastic basis of DNET [5], considering it to represent a hamartomatous anomaly with abnormal arrangements of normal neuronal and glial components. This is not a widely recognized perspective, with cases of malignant transformation [6, 7] and regrowth following subtotal resection [8] supporting the hypothesis that it is a true neoplasm.

Localized DNET is widely reported, but multifocal DNET remains an extremely rare entity, with seven previous instances in the literature (six with imaging). Associations with neurofibromatosis type 1 [9] and the Klinefelter (XXY) syndrome [10] have been reported, although our patient does not suffer from either condition and is presumed to have sporadic disease. Surrounding cortical dysplasia, as evident here, has previously been seen in association with multifocal DNET [3, 11]. Our patient presented with complex partial seizures of temporal lobe origin with one episode of secondary generalization, but is now well controlled on antiepileptic monotherapy. Evolution in symptoms has been reported in multifocal DNET [11], but it remains to be seen if the nature of seizures in our patient modifies further in future.

To our knowledge, intradural spinal cord lipomas have not previously been reported in a patient with DNET (multifocal or otherwise). Intradural spinal cord lipomas are rare, representing less than 1% of all spinal cord tumours, and are more commonly present in association with spinal dysraphism (not known to be the case in our patient). Intradural lipomas are generally regarded to arise during neurulation due to premature disjunction of neural ectoderm from cutaneous ectoderm (prior to neural tube closure). This allows mesenchyme access to the neural groove, in which it comes into contact with the primitive ependymal lining of the groove and subsequently develops into fat, indistinguishable from normal body fat. The process of neurulation begins at approximately the 15th day of development and terminates when the neural tube closes at between 24 days (cephalic end) and 27 days (caudal end) [12]. By contrast, while the developmental origins of multifocal DNET are uncertain, the presence of multiple bilateral nodules in a somewhat centrifugal pattern strongly suggests germinal cell origin, probably occurring during germinal proliferation or the migration process [11]. The mitotic activity/proliferation in the subependymal layers of the ventricles begins in approximately the 7th week of development followed by migration in the 8th week [13]. However, while these processes (neurulation and germinal matrix proliferation/migration) are embryologically distinct, a genetic link cannot be entirely excluded.

DNET can be recognized on MR imaging—as on histopathological analysis—from its glioneuronal element, with multiple T1-hypointense and T2-hyperintense “pseudocystic” areas of different sizes which return variable FLAIR (fluid-attenuated inversion recovery) signal [14]. These may have surrounding high signal on FLAIR separate from the glioneuronal element. Peritumoural edema, midline shift or significant contrast enhancement is not typical. MR spectroscopy may aid diagnosis, with findings including nonelevation of the ratio of choline-containing compounds to creatine, normal creatine peak and low N-acetylaspartate peak [15]. While unifocal DNET may be mistaken for low-grade glial tumours—hence may be far more common than initially thought—imaging findings in this case are fully consistent with previously reported cases of bilateral multifocal DNET with discrete nodules, and the authors believe that these represent pathognomonic features. Due to the indolent nature of the entity, no biopsy was performed; however, the imaging findings are thought to be fairly diagnostic of the entity. The presence of intradural lipomas in this patient is a unique presentation.
